# Functional macrophyte trait variation as a response to the source of inorganic carbon acquisition

**DOI:** 10.7717/peerj.12584

**Published:** 2021-12-01

**Authors:** Rafał Chmara, Eugeniusz Pronin, Józef Szmeja

**Affiliations:** Department of Plant Ecology, Faculty of Biology, University of Gdańsk, Gdańsk, Poland

**Keywords:** Carbon acquisition strategy, Leaf circularity, Leaf economic spectrum, Softwater lakes

## Abstract

**Background:**

This study aims to compare variation in a range of aquatic macrophyte species leaf traits into three carbon acquisition groups: HCO_3_^−^, free CO_2_ and atmospheric CO_2_.

**Methods:**

The leaf functional traits were measured for 30 species from 30 softwater lakes. Macrophyte species were classified into (1) free CO_2_, (2) atmospheric CO_2_ and (3) bicarbonate HCO_3_^−^ groups. In each lake we collected water samples and measured eight environmental variables: depth, Secchi depth, photosynthetically active radiation (PAR), pH of water, conductivity, calcium concentration, total nitrogen and total phosphorus. In this study we applied the RLQ analysis to investigate the relationships between species functional traits (Q) and their relationship with environmental variables (R) constrained by species abundance (L).

**Results:**

The results showed that: (1) Aquatic macrophytes exhibited high leaf trait variations as a response to different inorganic carbon acquisition; (2) Traits of leaves refer to the acquisition of carbon for photosynthesis and serve to maximise this process; (3) In the wide softwater habitat, macrophyte species exhibited an extreme range of leaf economic spectrum (leaf area, leaf dry weight and specific leaf area) and wide range of shape trait expressed as circularity; (4) Macrophyte leaf traits are the result of adaptation to carbon acquisition in ambient environment.

## Introduction

Aquatic macrophytes as non-taxonomic group (Bolton, 2016) comprise a wide range of growth forms and many classifications (Wiegleb, 1991; Wiegleb et al., 2015). Numerous growth forms of such plants are the manifestation of high phenotypic plasticity and adaptation to their environmental heterogeneity (Santamaría, 2002; Alahuhta et al., 2017). Morphological variations of aquatic plants are less variable than those of terrestrial plants (Maberly & Gontero, 2018). Due to underwater environment, macrophytes have limited access to carbon and experience reduced light levels (Pedersen, Colmer & Sand-Jensen, 2013). Moreover, submerged aquatic plants have a limited oxygen and free CO_2_ exchange between leaves and the environment (Mommer & Visser, 2005; Mommer et al., 2005).

The submerged aquatic plants use inorganic carbon for photosynthesis from water and/or sediment (Maberly & Spence, 1983; Raven, Osborne & Johnston, 1985; Keeley, 1998). When considering the location of carbon acquisition, it should be noted that it may come from the sediment collected by isoetids (Søndergaard & Sand-Jensen, 1979; Richardson et al., 1984), which are evergreen and well adapted to clear-water acidic lakes with low inorganic nutrients and carbon level (Arts, 2002).The next group of macrophytes (elodeids, charophytes, bryophytes and sphagnum mosses) takes carbon for the photosynthesis from the surrounding water and additionally from the sediments. Aquatic plants with floating leaves (excluding emergent macrophytes) might use atmospheric carbon dioxide (CO_2_) (Iversen et al., 2019). Thus, in this group we can find species which obligatorily use free CO_2_ and those that obligatorily use the HCO_3_^−^ for photosynthesis (Smith & Walker, 1980; O’Leary, 1988).

The form of inorganic carbon (HCO_3_^−^_,_ CO_2_) depends on the source (water, sediment, or air). In water the proportion between CO_2_ and another form of carbon depends on the pH of water. In the very acidic water (pH ≥ 4.3–5.6), the primary carbon source is CO_2_ dissolved in the water as a dissociated form of H_2_CO_3_. In almost neutral water (pH from about 6.5 to 7.5), the proportion of CO_2_ and HCO_3_^−^ is shifting to bicarbonates’ domination in the pH of water reaching about 8 (Iversen et al., 2019). That variation in the source of carbon acquisition and the possibility of using an appropriate form of carbon in the photosynthesis encouraged us to recognise how those different strategies are reflected in the functional indices of macrophytes.

The main theme of this work involves the functional traits of macrophytes. Therefore, it is worth noting that functional traits are defined as any morphological, anatomical or physiological characteristics of organisms at the individual level (Díaz et al., 2007; Pérez-Harguindeguy et al., 2013). According to Díaz et al. (2007), the functional diversity is a measure different from the taxonomic diversity, which takes into account the relative abundance of species in a community (Pla, Casanoves & Di Rienzo, 2012). It is important that the functional diversity should be based on characteristics of plant species in the lake. The functional trait is a feature influencing survival, such as reproduction and growth (Violle et al., 2007), plant height, plant longevity or specific leaf area (Lavorel & Garnier, 2002). In recent years, macrophyte functional traits have been examined in a number of studies (Fu et al., 2014; Chmara, Banaś & Szmeja, 2015; Chmara, Szmeja & Banaś, 2018; Zervas et al., 2019; Liu, Liu & Xing, 2021).

For all plants, including macrophytes, the leaf is an important organ, involved in the absorption and photochemical conversion of light energy, carbon uptake and synthesis of organic substances (Pulido et al., 2011). Aquatic leaf size and shape vary intraspecifically across species and environments (Dalla Vecchia, Villa & Bolpagni, 2020; Pierce et al., 2012). It is also worth noting that the leaf construction costs (measured as energy) are 1.5 times larger than the costs of stem (Griffin, 1994), which further justifies the choice of the leaf as an organ for analysing the functional traits of aquatic macrophytes. Thus, in this study, we decided to use a few leaf functional traits defined as leaf economic spectrum (*sensu* Wright et al., 2004): leaf area (LA mm^2^), leaf dry weight (LDW mg), specific leaf area (SLA mm^2^ mg^−1^) and shape trait circularity should be listed. We suspect that those traits will differentiate in relation to the carbon acquisition strategy of 30 macrophytes species investigated by us. With no doubt the shape traits such as circularity and leaf area differentiate along macrophytes ecological groups from nympheids to bryophytes and might be related also to different carbon uptake strategies.

The aim of this work is to analyse the leaf functional traits of macrophytes especially in softwater lakes with isoetids, based on leaf traits. The North European softwater lakes are defined according to physico-chemical conditions given by OECD (1982) and recommendations by Moss, Johnes & Phillips (1996). They are lakes with Ca^2+^ < 3 mg L^−1^ and very low alkalinity of water and typified by aquatic plant species which are more or less carbon-limited (Murphy, 2002; Pulido et al., 2011).

There is no doubt that macrophytes compensate environmental constraints with various morphological, anatomical and physiological adaptation to maximise inorganic carbon uptake in the environment (Yin et al., 2017; Maberly & Gontero, 2018). Based on these studies, as well as our research of plants in lakes, we hypothesise that their responses to the source of inorganic carbon acquisition are a manifestation of the species’ life strategies resulting from leaf morphology. The aim of our work is to determine the relationship between the inorganic carbon acquisition for photosynthesis by macrophytes with the morphology of their leaves. Here, we focus on the comparison of leaf traits of 30 macrophyte species into three carbon acquisition strategies.

## Materials and Methods

### Study site and field sampling

The study was performed in north-western Poland, in the Pomeranian Lakeland (53°48′51.1N, 17°38′00.9E) in 30 softwater lakes in June and August from 2014 to 2020. All lakes belong to softwater lake types and their environmental conditions represent a wide spectrum of softwater habitat, water acidity (pH 4.1–7.9) and calcium concentration (1.0–18.6 mg/L). The geographic coordinates and morphometric features of these lakes were presented in our previous study (Chmara, Szmeja & Robionek, 2019). To investigate species abundance the aquatic macrophytes were sampled in 30 lakes along depth zones in a transect, perpendicularly to the shoreline. In each of these lakes, one transect was delineated. At each transect, a diver randomly collected macrophyte samples until the maximum macrophyte occurrence depth. Macrophyte abundance was expressed as a cover-plant sample (squares with area = 0.1 m^2^). For study of several protected macrophyte species, permission of Regional Director for Environmental Protection in Gdańsk, Poland (No. RDOŚ-Gd-WZG.6400.92.2020.AB.2) was obtained. A total of 145 depth zones in 30 transects were designated to determine macrophytes presence and abundance.

### List of macrophyte species divided into inorganic carbon acquisition

The information about species inorganic carbon acquisition was collected from the scientific literature: aquatic angiosperms (Maberly & Madsen, 2002; Iversen et al., 2019); bryophytes (Riis & Sand-Jensen, 1997) and charophytes (Baastrup-Spohr et al., 2015). Finally, each species was assigned to one of three inorganic carbon groups. A total of 30 species were selected and included in the statistical analyses.

### Measurement of leaf traits

We measured four leaf traits of 30 macrophyte species: leaf area (LA mm^2^), leaf dry weight (LDW mg), specific leaf area (SLA mm^2^ mg^−1^) and shape trait circularity [4π(area × perimeter^−2^)]. Leaves were collected in June and August from 2016 to 2020 in 30 softwater lakes. Subsequently, 30 healthy leaves were collected from three to five individuals of each aquatic macrophyte species. Plant species names were checked according to The Plant List (http://www.theplantlist.org/). Leaf traits were determined following standardised methods of Pérez-Harguindeguy et al. (2013). We made measurements of charophytes and bryophytes functional traits. For measurement of charophytes traits we used the branchlets which are equivalents of the leaves of higher plants (Soulié–Märsche, 1999). For measurement of leaf traits, each leaf was photographed while fresh. Photos of bryophyte leaves and branchlets (charophytes) were taken using a Nikon Coolpix MDC Lens camera and Nikon SMZ 1500 stereomicroscope, Tokyo, Japan. Measurements of leaf area were calculated using ImageJ software. Leaf mosses and branchlets of the charophytes were weighed with a precision balance at 0.01 mg resolution. Mosses’ and charophytes’ specific leaf area was calculated as the leaf area (mm^2^) per unit of leaf dry mass (mg), determined with a precision scale. Leaves of vascular plants were assessed by using a standard flatbed scanner for leaf area; circularity was measured by means of ImageJ ver. 1.46 (http://imagej.nih.gov/ij) open-source software. Circularity was calculated according to Krieger (2014), circularity is mathematically constrained to range from 0 for a line to 1 for a circle.

All leaves were dried at 80 °C for 48 h, and the final dry mass was measured. Specific leaf area was calculated as the leaf area (mm^2^) per unit of leaf dry mass (mg). Species were classified into (1) free CO_2_, (2) atmospheric CO_2_ and (3) bicarbonate HCO_3_^−^ groups based on the previous studies (Iversen et al., 2019; Maberly & Madsen, 2002).

Detailed information on the qualitative values of aquatic plants traits was archived in the AQUA-PLANT-TRAIT-UGDA DATABASE in the Department of Plant Ecology, University of Gdańsk.

### Environmental data

In each lake we collected water samples and measured eight environmental variables during the vegetation seasons (in June and August) from 2016 to 2020. The samples were collected by SCUBA divers. A total of 465 water samples were collected in the depth zones; each sample containing 500 ml. The following environmental factors were determined in the depth zones: depth (m), visibility (m), photosynthetically active radiation (PAR, in % of the light reaching the water surface), pH of water, conductivity (µS/cm), calcium concentration (mgCa^2+^ L^−1^), total nitrogen (mgN L^−1^) and total phosphorus (mgP L^−1^). The measurements were performed according to Eaton et al. (2005). PAR was measured in the depth zones with 0.5 m intervals by means of Licor LI–250 Light Meter.

### Data analysis

Macrophyte species were divided into three carbon acquisition groups: (1) free CO_2_, (2) atmospheric CO_2_; (3) bicarbonate HCO_3_^−^. We assessed differences in the functional traits (LA, LDW, SLA, circularity) into carbon acquisition groups using basic statistics and we applied coefficients of variations formula (CV = traits (SD)/traits (mean) × 100%, where SD - standard deviation). To test traits variations into three carbon acquisition groups, non-metric multidimensional scaling (nMDS) was performed. The nMDS algorithm was then used as Bray–Curtis distances between samples. The nMDS analysis was run in PAST ver. 4.05. To compare the values of leaf traits grouped into carbon acquisition, we used the non-parametric Kruskal–Wallis test, followed by Dunn’s multiple comparisons *post hoc* test. All trait values were log_10_-transformed.

The RLQ analysis (R-mode; Q-mode; and L-link between R and Q) was applied to investigate the relationships between species functional traits (Q) and environmental variables (R) constrained by species abundance (L) (Dolédec et al., 1996; Stefanidis & Papastergiadou, 2019). This method, since its development, is widely applied in functional trait studies that combine separate analyses on multiple datasets to identify the relationships between traits and environmental variables, weighed by the abundances of species (Dolédec et al., 1996; Stefanidis & Papastergiadou, 2019; Zervas et al., 2019). Similarly to the method procedure described by Stefanidis & Papastergiadou (2019), the first step for RLQ analysis implementation is to create the ordinations analysis on each table, R, L and Q separately. Table R with the environmental variables is limited only by quantitative data; thus, the Principal Component Analysis (PCA) was applied as was pointed by Stefanidis & Papastergiadou (2019). As Zervas et al. (2019) explained that the Correspondence Analysis (CA) was performed on species data in table L. Next, the data analysis procedure for the functional trait table, Q Hill and Smith analysis (Hill & Smith, 1976) were used. The fundamental assumptions of this RLQ method are ordination positioning based on results of CA analysis depended on scores of sites and species data from table L, next the row weights obtained from PCA and in the end the results values of Hill and Smith analysis based on data from table Q (Zervas et al., 2019). The maximum covariance between data of the functional traits and related to them, the environmental variables are shown on the obtained graphs and reports corresponding to those graphs in the R software environment (Dray et al., 2014). Following Stefanidis & Papastergiadou (2019) data analysis procedure, this relationship’s overall significance was tested using a global Monte-Carlo test depending on the rows from table R and those of table Q. As Stefanidis & Papastergiadou (2019) explained, the contribution of each trait and environmental parameter to total inertia was used and presented as a measure of relative importance and helped us to identify the most important traits and environmental factors. All analyses related to the RLQ method were performed by using the *ade4* package library (Dray & Dufour, 2007) in R environment version 4.0.2 (R Core Team, 2020).

## Results

### Differences in macrophyte leaf traits between carbon acquisition groups

In total, functional trait data of 30 macrophyte species was collected, species were grouped into three carbon acquisition groups: free CO_2_ (12 species), atmospheric CO_2_ (3 species) and bicarbonate HCO_3_^−^ (15 species; Table 1). Within the free CO_2_ group we observed mainly mosses (including *Sphagnum* mosses) and isoetids, but in the atmospheric CO_2_ group only floating-leaved species. Within the bicarbonate acquisition group we noted charophytes and vascular plants belonging to different growth-forms and leaf types. Interspecific traits variations ranged broadly, the means of LA, LDW, SLA and circularity were 0.85–4,095.7 mm^2^, 0.003–36,120.5 mg, 10.9–342.4 mm^2^ mg^−1^, 0.005–0.920, respectively. LA varied among carbon acquisition groups from 81.0 mm^2^ in the free CO_2_ group to 7,473.4 mm^2^ in the atmospheric CO_2_ group, LDW ranged from 2.5 mg in free CO_2_ group to 751.5 mg in the atmospheric CO_2_ group and SLA ranged from 16.9 mm^2^ mg^−1^ in the atmospheric CO_2_ group to 172.1 mm^2^ mg^−1^ in free CO_2_ group (Table 2). Table 2 shows high interspecific variability among macrophyte functional traits, with coefficients (CV) of variation ranging from 19.96% to 264.45%. For circularity and SLA, the CV was lower than that of LA and LDW.

**Table 1:** List of macrophyte species divided into carbon acquisition groups, mean cover, growth-form and leaf type.

Species	Carbon acquisition groups			Cover % mean ± s.d.	Growth-form	Leaf type
	Free CO_2_	Atmospheric CO_2_	HCO_3_^−^			
*Drepanocladus sordidus* (Müll. Hal.) Hedenäs	•	–	–	16.03 ± 13.83	C/B	LT3
*Eleocharis acicularis* (L.) Roem. & Schult	•	–	–	32.51 ± 30.80	I	LT1
*Fontinalis antipyretica* Hedw.	•	–	–	16.51 ± 14.63	C/B	LT3
*Fontinalis dalecarlica* Bruch & Schimp.	•	–	–	24.88 ± 24.11	C/B	LT3
*Isoëtes lacustris* L.	•	–	–	48.17 ± 33.97	I	LT1
*Juncus bulbosus* L.	•	–	–	25.27 ± 28.11	I	LT1
*Littorella uniflora* (L.) Asch.	•	–	–	33.91 ± 30.02	I	LT1
*Lobelia dortmanna* L.	•	–	–	29.65 ± 25.88	I	LT1
*Sparganium angustifolium* F. Michx.	•	–	–	4.37 ± 4.28	V	LT3
*Sphagnum cuspidatum* Ehrh. ex Hoffm.	•	–	–	41.53 ± 25.32	C/B	LT3
*Sphagnum denticulatum* Brid.	•	–	–	30.09 ± 26.91	C/B	LT3
*Warnstorfia exannulata* (Schimp.) Loeske	•	–	–	20.64 ± 21.36	C/B	LT3
*Nuphar lutea* (L.) Sibth. & Sm.	–	•	–	22.19 ± 23.29	N	LT3
*Persicaria amphibia* (L.) Delalbre	–	•	–	10.39 ± 9.69	N	LT3
*Potamogeton natans* L.	–	•	–	22.23 ± 20.76	N	LT3
*Ceratophyllum demersum* L.	–	–	•	13.61 ± 15.79	PL	LT2
*Chara virgata* Kützing	–	–	•	46.59 ± 35.07	C/CH	LT2
*Chara globularis* Thuiller	–	–	•	35.62 ± 30.19	C/CH	LT2
*Elodea canadensis* Michx.	–	–	•	17.36 ± 16.91	P	LT3
*Luronium natans* (L.) Raf./submerged leaves/	–	–	•	37.69 ± 30.52	I	LT3
*Myriophyllum alterniflorum* DC.	–	–	•	21.84 ± 22.12	M	LT2
*Myriophyllum spicatum* L.	–	–	•	11.35 ± 5.32	M	LT2
*Nitella flexilis* (L.) AG.	–	–	•	21.83 ± 26.34	C/CH	LT2
*Nitellopsis obtusa* (Desvaux) Groves	–	–	•	11.97 ± 12.70	C/CH	LT2
*Potamogeton crispus* L.	–	–	•	15.97 ± 9.81	P	LT3
*Potamogeton gramineus* L. /submerged leaves/	–	–	•	11.31 ± 12.56	P	LT3
*Potamogeton obtusifolius* Mert. & W.D.J. Koch	–	–	•	7.28 ± 11.40	P	LT3
*Potamogeton x nitens* Weber	–	–	•	32.00 ± 27.56	P	LT3
*Ranunculus reptans* L.	–	–	•	15.85 ± 21.32	I	LT3
*Stuckenia pectinata* (L.) Börner	–	–	•	21.38 ± 15.42	P	LT2

**Note:**

(1) Growth-form, PL, Pleustophyte; C/CH, Cryptogam/Charophyta; C/B, Cryptogam/Bryophyta; I, Isoetid; P, Potamid; M, Myriophyllid; N, Nymphaeid; V, Vallisnerid; (2) Leaf type, LT1, tubular; LT2, capillary; LT3, flat-leaf.

**Table 2:** Leaf trait values in the acquisition carbon groups. SD, standard deviation; range, min-max. values; CV, coefficient of variation.

Trait	Mean	SD	Range	CV (%)
Free CO_2_				
LA (mm^2^)	81.01	193.62	[0.33–2,373.95]	239.04
LDW (mg)	2.51	6.64	[0.002–80.0]	264.45
SLA (mm^2^ mg^−1^)	172.09	130.17	[11.77–504.33]	73.64
Circularity	0.25	0.19	[0.018–0.714]	73.76
Atmospheric CO_2_				
LA (mm^2^)	7,473.39	14,746.64	[259.5–61,191]	197.32
LDW (mg)	751.50	1834.88	[5.7–9,074]	244.16
SLA (mm^2^ mg^−1^)	16.95	5.02	[5.97–36.07]	29.61
Circularity	0.66	0.13	[0.31–0.92]	18.96
Bicarbonate HCO_3_^−^				
LA (mm^2^)	144.14	163.23	[3.95–779.13]	113.24
LDW (mg)	2.94	3.54	[0.1–21.4]	120.43
SLA (mm^2^ mg^−1^)	68.61	52.11	[5.97–227.4]	75.95
Circularity	0.24	0.22	[0.005–0.77]	92.41

**Figure 1:**
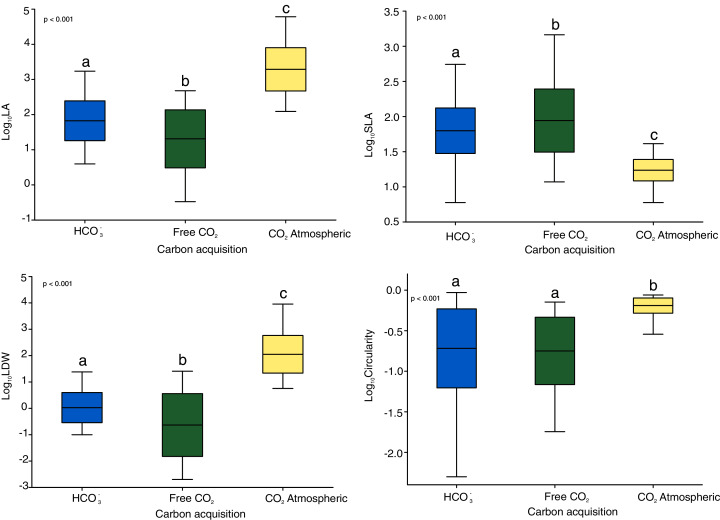
Functional and shape leaf traits in different carbon acquisition. LA: leaf area, LDW: leaf dry weight content, SLA: specific leaf area. Values are log-transformed, Whiskers are standard deviations. Different letters indicate significant differences between carbon acquisition groups for a given trait. Letters denote the result of pairwise comparisons (Dunn’s test of multiple comparisons of independent samples). Significant levels are showed by *p* value: *p* < 0.01.

**Figure 2:**
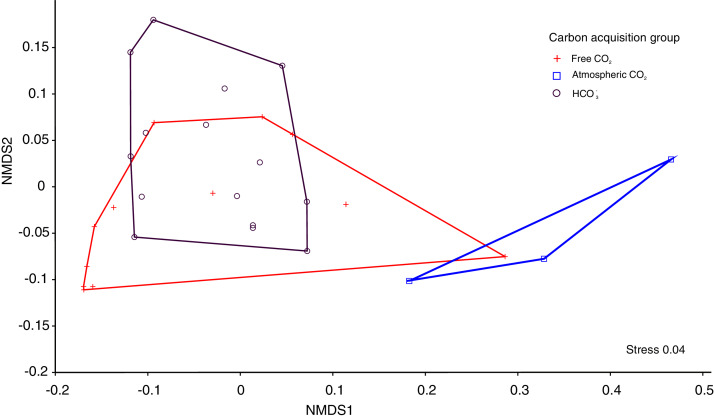
The nMDS ordination leaf traits of 30 macrophytes in 30 softwater lakes.

The Kruskal-Wallis tests showed significant differences among the four functional traits in relation to carbon acquisition groups (LA, SLA, LDW, Fig. 1, *p* < 0.001). LA, LDW and circularity in the free atmospheric CO_2_ group were significantly higher compared to the other groups (values of Kruskal–Wallis test: *χ*^2^ = 192.0, *df* = 2, *p* < 0.001; *χ*^2^ = 199.2, *df* = 2, *p* < 0.001) LA and LDW of bicarbonate acquisition group varied not that much as in the free CO_2_ and atmospheric CO_2_ groups. However, SLA in the free CO_2_ group showed relatively lower log-values than the other groups. Circularity in the free CO_2_ and bicarbonate groups did not differ significantly (*p* = 0.48, Fig. 1). Additionally, the nonmetric multidimensional scaling (nMDS) analysis showed functional trait differences among carbon acquisition groups (Fig. 2), ANOSIM statistics of the assessed groups: *R* = 0.42, *p* = 0.002. Generally, nMDS diagram illustrates that the atmospheric CO_2_ species were the least overlapping in the diagram, while the other two groups showed more similarities.

### Environmental effects on leaf traits

**Figure 3:**
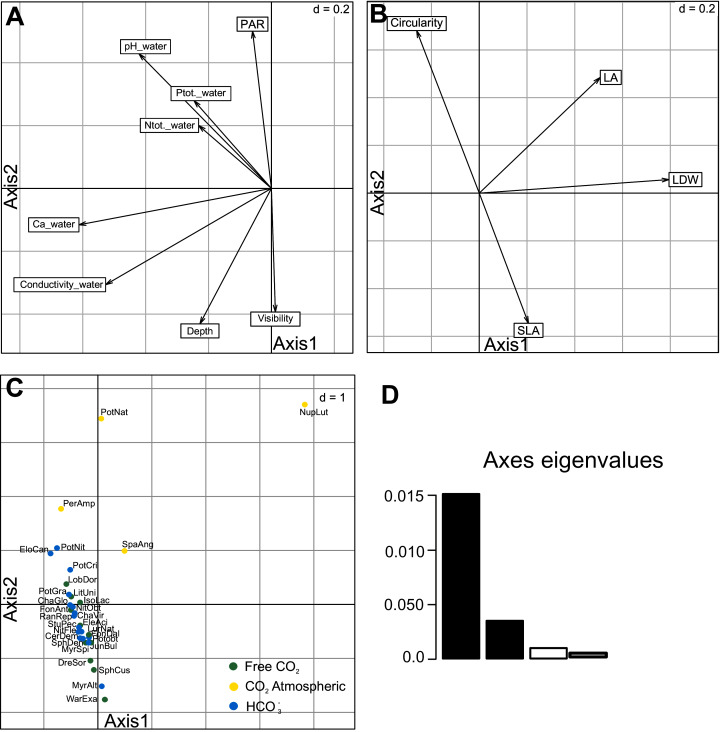
Results of the first and second axes of RLQ analysis. Environmental variables (A), traits (B), species scores (C) and eigenvalues first two axes (D). Species code abbreviation can be found in Table S2.

The RLQ analysis explained very well the cross-covariance between species functional traits and environmental variables. First two RLQ axes explained 94.40% of the total inertia (1st axis = 76.79%; 2nd axis = 17.61%; Fig.1D; Table 3, Table S1). The first axis with environmental variables differentiated sites with higher conductivity, calcium ion concentration and pH of water (Fig. 3A). Among the environmental variables, Ca^2+^ concentration, conductivity and visibility have a higher share in the total inertia (Table 4). In the species functional traits, the first axis was positively correlated with the measured leaf dry weight (LDW), leaf area (LA). The second axis was correlated with the SLA and circularity which were negatively correlated to each other (Fig. 3B). Regarding the macrophyte functional traits, LA and LDW (Table 4) have the highest share in the total inertia. Species were also discriminated against each other according to these two axes (Fig. 3C). All investigated species were placed along the second axis where *Sphagnum* mosses and *Warnstorfia exannuata* were placed at the bottom, the species from elodeids and isoetids group dominated in the centre. At the top, the species with floating leaves were positioned. An exception to the mentioned rule was noted for *Nuphar lute*a whose position was more related to the first axis, and it was placed in the right-upper corner (Fig. 3C).

**Table 3:** Summary of the RLQ analysis. The table presents reports of the eigenvalues (and percentage of total co-inertia) for the two main axes, covariance and correlation (and percentage of total correlation) with the CA on matrix L (species), and projected inertia (and percentage of total inertia) with the R (the environmental variable matrix) and Q the (species traits matrix) matrices. The ratio of inertia and co-inertia for R and Q as well as the ratio of correlation of L corresponded to Axis 1 and Axis 2 are also presented.

RLQ analysis	Axis 1 (%)	Axis 2 (%)
RLQ eigenvalues	0.115 (76.79%)	0.026 (17.61%)
Covariance	0.34	0.16
Correlation L (sp)	0.19 (16.11%)	0.10 (12.39%)
Projected inertia R (env)	3.24 (40.54%)	1.94 (24.21%)
Projected inertia Q (trait)	2.63 (65.75%)	0.82 (20.51%)
Rtio of inertia and co-inertia R (env)	0.84	0.76
Rtio of inertia and coinertia Q (trait)	0.44	0.92
Rtio of correlations L (sp)	0.25	0.16

**Table 4:** Percentage contribution of the environmental variables and functional traits to the RLQ analysis.

Environmental variable	Contribution to total inertia (%) Axis 1	Contribution to total inertia (%) Axis 2	Macrophyte trait	Contribution to total inertia (%) Axis 1	Contribution to total inertia (%) Axis 2
Ca_w	22.98	7.97	LA	35.14	2.25
Conductivity_w	20.75	3.42	LDW	30.04	6.00
Visibility	13.85	20.81	Circularity	24.69	2.43
pH_w	13.05	8.86	SLA	10.13	89.31
Ptot._w	12.48	5.72			
Ntot._w	12.40	8.23			
Depth (m)	2.84	22.31			
PAR (%)	1.65	22.68			

## Discussion

### Response of leaf traits to carbon acquisition

The pH of water of the sampled lakes was between 4.1 and 7.9, which represents the full spectrum of softwater habitat. Under these conditions macrophytes take up three inorganic carbon forms (free carbon dioxide, atmospheric carbon dioxide and bicarbonate). Our results showed significant differences in macrophyte traits as a response to the source of inorganic carbon acquisition (Table 1, Figs. 1 and 2). Recent studies reported trait differences in growth-forms (Pierce et al., 2012), native/alien aquatic plants species (Lukács et al., 2017) and leaf types (Liu, Liu & Xing, 2021). We found no reports of functional traits of macrophytes with different inorganic carbon acquisition groups. We found an extreme range of leaf economic spectrum (leaf area, leaf dry weight and specific leaf area) and wide range of shape trait, circularity (Table 2). Among these groups we found high LA and LDW interspecific variations expressed as coefficients of variations. The highest values of specific leaf area were found in free CO_2_ acquisition groups, especially *Sphagnum* mosses and *Warnstorfia exannulata*. Leaves of these species are small and extremely thin with typical one-cell thickness. The one-cell thick leaves permit the light and free CO_2_ to reach photosynthetic cells directly (Glime, 2014). Furthermore, the consequence of high SLA is rapid and economic acquisition of CO_2_ as a typical trade-off between rapid acquisition and conservation of resources (Wright et al., 2004).

In contrast, macrophytes using atmospheric CO_2_ differ in leaf functional traits compared to previous groups. Leaves of aquatic plants that float, have stomata at upper surface (Rudall & Knowles, 2013). Moreover, they tend to decrease SLA and increase LDW and LA, and are more oval. Low CV of circularity indicated small shape differences. Leaf area trait of emergent macrophytes correlated with nutrient concentration (Garnier et al., 2001; Wright, Reich & Westoby, 2001). The RLQ analysis showed that *Nuphar lutea* leaf traits related to the first axis correlated with conductivity, calcium ion concentration and pH of water (Table 4, Fig. 3C). In our study area, *Nuphar lutea* occurred most often in acidic softwater lakes and softwater-lobelia lakes with acidophytic mosses, where it forms heterophyllous leaves, floating leaves with long petioles and submersed leaves with short petioles. The functional traits of these leaves are different; they take up free carbon dioxide and atmospheric carbon dioxide.

In our study, the number of 15 species (50%) in the bicarbonate acquisition group is close to 44% of the total 131 investigated submerged aquatic plants with the capability of using HCO_3_^−^ investigated by Iversen et al. (2019). It should be noted that those plant species use bicarbonate as a carbon source but in the conditions where this source is limited they might also use CO_2_, which is sometimes not strictly pointed out in the available literature (Maberly & Madsen, 2002; Iversen et al., 2019). Our study was performed within a huge range of the pH of water (from 4.1 to 7.9); thus, the species we investigated had the suitable conditions to use both above-mentioned carbon forms for the process of photosynthesis. Our study showed that the LA and LWD functional traits of bicarbonate acquisition group varied not that much as in the free CO_2_ and atmospheric CO_2_ groups (Table 2, Fig. 1), which might be related to the adaptation to permanently submerged conditions and ability to considerably take up carbon and other nutrients mainly from water (Maberly & Gontero, 2018). Our study confirmed that low specific leaf area in aquatic macrophytes might reflect the dominance of bicarbonate users (Lukács et al., 2019). Moreover, the CV of circularity was the highest in this group (Table 2), which is related to the greater variability of different types of macrophytes species (charophytes and vascular plants belonging to different growth forms and leaf types).

### Differences in ecological strategies between carbon acquisition groups

Our study found high traits variations in the carbon acquisition groups. These findings showed the rapid carbon acquisition strategy of macrophyte species in softwater lakes. We agree with the previous study showing that aquatic plants exhibit numerous strategies to increase carbon uptake (Maberly & Gontero, 2018). This diversity explains well the three carbon acquisition strategies: avoidance, exploitation and amelioration (*sensu* Klavsen, Madsen & Maberly, 2011). We investigated that macrophytes follow these carbon acquisition strategies in the softwater lakes. Firstly, mosses with small leaves, extreme thin and high SLA live and grow in microhabitats with locally high free carbon dioxide and employ the avoidance strategies. Secondly, isoetids follow the exploitation strategies which involve morpho-anatomical features (lacunae in leaves and roots, thick cuticles) to higher concentrations of CO_2_. Thirdly, these strategies also include floating-leaved species (*e.g. Nuphar lutea*, *P. amphibia* and *P. natans*) with access to CO_2_ in the atmosphere. Furthermore, the amelioration strategies with energy-requiring processes utilise bicarbonate as a source of carbon. For water with air-equilibrium carbon dioxide concentration, the energy cost of photorespiration with diffusive carbon dioxide entry can exceed that of a carbon dioxide concentrating mechanism (often involving bicarbonate entry), which can largely suppress Rubisco oxygenase and hence photorespiration (Raven, Beardall & Giordano, 2014). In our study, amelioration strategies include numerous growth-forms: potamids, myriophyllids and charophytes (Table 1).

### Knowledge gaps

The available scientific data on aquatic plant shape traits is incomplete, non-representative, mainly descriptive and has never been evaluated. A recent study based on the two leaf-shape types of macrophytes (flat-leaf type, needle-leaf type) showed different adaptive strategies to lake eutrophication and water depth-leaf shape relationships (Liu, Liu & Xing, 2021). Other studies emphasised that leaf shape, as an important phenotypic trait, can reflect the adaptation of macrophytes to environmental constraints (Maberly & Gontero, 2018; Pierce et al., 2012).

Our study indicated significant differences in leaf shapes as a response to carbon acquisition and higher contribution to total inertia of the first axis in RLQ analysis (Table 4, Fig. 3B). Furthermore, these findings are based on the trait metric expressed as circularity showing for the first time that aquatic macrophytes represent almost full spectrum of circularity metric (0.018–0.92). We propose circularity as a leaf-shape trait quick and easy to measure. It would also be interesting to assess relationships between leaf circularity and other traits into carbon acquisition groups.

## Conclusions

Our study found that:

1. Aquatic macrophytes showed high leaf trait variations as a response to different inorganic carbon acquisition
2. Traits of leaves refer to the acquisition of carbon for photosynthesis and serve to maximise this process.
3. In the wide softwater habitat, macrophyte species exhibited an extreme range of leaf economic spectrum (leaf area, leaf dry weight and specific leaf area) and wide range of shape trait expressed as circularity.
4. Macrophyte leaf traits are the result of adaptation to carbon acquisition in ambient environment. Linkages between leaf trait-carbon acquisition will be helpful our understanding of aquatic macrophytes adaptations.

## Supplemental Information

10.7717/peerj.12584/supp-1 Supplemental Information 1 RLQ analysis summary.

10.7717/peerj.12584/supp-2 Supplemental Information 2 Macrophyte species and their abbreviation code.

10.7717/peerj.12584/supp-3 Supplemental Information 3 The R, L and Q matrices used for the RLQ analysis.

## Additional Information and Declarations

### Field Study Permissions

The following information was supplied relating to field study approvals (*i.e*., approving body and any reference numbers): The Regional Director for Environmental Protection in Gdańsk permitted the study (RDOŚ-Gd-WZG.6400.92.2020.AB.2).

### Data Availability

The following information was supplied regarding data availability: The dataset is available in the Supplemental File.
