# Supplementary material for: Functional macrophyte trait variation as a response to the source of inorganic carbon acquisition
Source: PeerJ. 2021 Dec 1;9:e12584. doi: 10.7717/peerj.12584 (PMC8643105; doi:10.7717/peerj.12584)
Supplement: Supplemental Information 1 [file peerj-09-12584-s001.docx]

Chmara R*., Pronin E., Szmeja J. Functional macrophyte trait variation as a response to the source of inorganic carbon acquisition

Table S1. RLQ analysis summary.

Class: rlq dudi

Call: rlq(dudiR = acpR.aravo, dudiL = afcL.aravo, dudiQ = acpQ.aravo,

scannf = FALSE)

Total inertia: 0.1503

Eigenvalues:

Ax1 Ax2 Ax3 Ax4

0.115450 0.026481 0.007338 0.001078

Projected inertia (%):

Ax1 Ax2 Ax3 Ax4

76.7890 17.6133 4.8807 0.7169

Cumulative projected inertia (%):

Ax1 Ax1:2 Ax1:3 Ax1:4

76.79 94.40 99.28 100.00

Eigenvalues decomposition:

eig covar sdR sdQ corr

1 0.11545006 0.3397794 1.653234 1.081281 0.1900747

2 0.02648112 0.1627302 1.099338 1.410380 0.1049545

Inertia & coinertia R (acpR.aravo):

inertia max ratio

1 2.733183 3.243542 0.8426538

12 3.941726 5.180119 0.7609335

Inertia & coinertia Q (acpQ.aravo):

inertia max ratio

1 1.169168 2.630024 0.4445465

12 3.158340 3.450586 0.9153053

Correlation L (afcL.aravo):

corr max ratio

1 0.1900747 0.7552896 0.251658

2 0.1049545 0.6625286 0.158415

summary(acpQ.aravo)

Class: pca dudi

Call: dudi.pca(df = Q1, row.w = afcL.aravo$cw, scannf = FALSE)

Total inertia: 4

Eigenvalues:

Ax1 Ax2 Ax3 Ax4

2.63002 0.82056 0.48009 0.06933

Projected inertia (%):

Ax1 Ax2 Ax3 Ax4

65.751 20.514 12.002 1.733

Cumulative projected inertia (%):

Ax1 Ax1:2 Ax1:3 Ax1:4

65.75 86.26 98.27 100.00

summary(afcL.aravo)

Class: coa dudi

Call: dudi.coa(df = R1, scannf = FALSE)

Total inertia: 3.541

Eigenvalues:

Ax1 Ax2 Ax3 Ax4 Ax5

0.5705 0.4389 0.3611 0.2752 0.2466

Projected inertia (%):

Ax1 Ax2 Ax3 Ax4 Ax5

16.110 12.396 10.199 7.771 6.964

Cumulative projected inertia (%):

Ax1 Ax1:2 Ax1:3 Ax1:4 Ax1:5

16.11 28.51 38.70 46.48 53.44

(Only 5 dimensions (out of 29) are shown)

summary(acpR.aravo)

Class: mix dudi

Call: dudi.hillsmith(df = L1, row.w = afcL.aravo$lw, scannf = FALSE)

Total inertia: 8

Eigenvalues:

Ax1 Ax2 Ax3 Ax4 Ax5

3.2435 1.9366 1.1031 0.7796 0.4416

Projected inertia (%):

Ax1 Ax2 Ax3 Ax4 Ax5

40.544 24.207 13.789 9.745 5.520

Cumulative projected inertia (%):

Ax1 Ax1:2 Ax1:3 Ax1:4 Ax1:5

40.54 64.75 78.54 88.29 93.81

(Only 5 dimensions (out of 8) are shown)
