# Supplementary material for: Functional macrophyte trait variation as a response to the source of inorganic carbon acquisition
Source: PeerJ. 2021 Dec 1;9:e12584. doi: 10.7717/peerj.12584 (PMC8643105; doi:10.7717/peerj.12584)
Supplement: Supplemental Information 2 [file peerj-09-12584-s002.docx]

Chmara R*., Pronin E., Szmeja J. Functional macrophyte trait variation as a response to the source of inorganic carbon acquisition

Table S2. Macrophyte species and their abbreviation code.

| Abbreviation code | Species |
| --- | --- |
| CerDem | *Ceratophyllum demersum* |
| CharVir | *Chara virgata* |
| CharGlob | *Chara globularis* |
| DreSor | *Drepanocladus sordidus* |
| EleAci | *Eleocharis acicularis* |
| EloCan | *Elodea canadensis* |
| FonAnt | *Fontinalis antipyretica* |
| FonDal | *Fontinalis dalecarlica* |
| IsoLac | *Isoëtes lacustris* |
| JunBul | *Juncus bulbosus* |
| LitUni | *Littorella uniflora* |
| LobDor | *Lobelia dortmanna* |
| LurNat | *Luronium natans* |
| MyrAlt | *Myriophyllum alterniflorum* |
| MyrSpi | *Myriophyllum spicatum* |
| NitFle | *Nitella flexilis* |
| NitObt | *Nitellopsis obtusa* |
| NupLut | *Nuphar lutea* |
| PerAmp | *Persicaria amphibia* |
| PotCri | *Potamogeton crispus* |
| PotGra | *Potamogeton gramineus* |
| PotNat | *Potamogeton natans* |
| PotObt | *Potamogeton obtusifolius* |
| PotNit | *Potamogeton x nitens* |
| RanRep | *Ranunculus reptans* |
| SpaAng | *Sparganium angustifolium* |
| SphCus | *Sphagnum cuspidatum* |
| SphDen | *Sphagnum denticulatum* |
| StuPec | *Stuckenia pectinata* |
| WarExa | *Warnstorfia exannulata* |
